# Long-Term Functional and Psychosocial Consequences and Health Care Provision after Traumatic Brain Injury

**DOI:** 10.1155/2016/2678081

**Published:** 2016-03-10

**Authors:** Nada Andelic, Solrun Sigurdardottir, Juan Carlos Arango-Lasprilla, Alison K. Godbolt

**Affiliations:** ^1^Department of Physical Medicine and Rehabilitation, Oslo University Hospital, 0424 Oslo, Norway; ^2^Sunnaas Rehabilitation Hospital, 1450 Nesoddtangen, Norway; ^3^Research Centre for Habilitation and Rehabilitation Models and Services (CHARM), Institute of Health and Society, Faculty of Medicine, University of Oslo, 0318 Oslo, Norway; ^4^IKERBASQUE-Basque Foundation for Science, 48007 Bilbao, Spain; ^5^University Department of Rehabilitation Medicine, Danderyd Hospital, Karolinska Institute, 18288 Stockholm, Sweden

Traumatic brain injury (TBI) has a wide range of severity ranging from concussion to severe brain injury and death [1]. A wide body of literature has documented adverse outcomes in cognitive, behavioral, emotional, and physical functioning after TBI, as well as increased risk for poor family and social functioning [2–5]. TBI presents a major challenge to health care systems because of residual functional impairments along with difficulties with activities of daily living, education, community integration, and employment [6–8]. To date, few studies have investigated these issues using longitudinal and mixed method research designs. Studies from different countries are required to allow a better understanding of international differences, cultural aspects, and patients' needs for health care services and additional support [9, 10].

The aim of this special issue is to present international multidisciplinary research on long-term functional and psychosocial consequences of TBI in populations of adolescents, adults, and the elderly. More specifically, authors of this issue addressed methods for the development of assessment tools in TBI, neurophysiology of cognitive capacity, cognitive, executive, emotional, behavioral, and vocational functioning, and health-related quality of life after TBI. It is our goal that the articles published in this issue will contribute to a better understanding of the long-term consequences and health care needs after TBI.

The development of culturally meaningful assessment tools for the measurement of outcomes and needs in TBI has been limited, which may in part be due to a limited focus on cultural aspects of TBI in international classification systems. Researchers from New Zealand, H. Elder and P. Kersten, used Whakawhiti korero, an indigenous research method that emphasizes discussion and negotiation, in the development of a cultural needs assessment tool for Māori traumatic brain injury. This method may have wider applicability in other fields, such as mental health and addiction services, to ensure a robust process of outcome measurement and needs assessment.

Accurate assessment of level of awareness and cognitive capacity of patients in a state of disordered consciousness after severe TBI is of utmost importance for rendering a correct diagnosis and designing treatment plans. S. L. Hauger et al. from Norway employed electrophysiological approaches to assess residual cognitive capacity using two tasks that differed as a function of cognitive load and stimulus type in this unique patient population. The findings revealed that an active task performed by the patients was robust in probing for volitional cognitive capacity, indicating that this can be an important tool for providing diagnostic information for patients in a minimally conscious state.

Suffering a TBI during adolescence may not only cause TBI-related impairments but also disrupt the adolescent's process of development towards an independent life as an adult. Thus adolescent TBI brings with it special issues and as such a research focus on this age group is welcomed. K. Doser et al. from Denmark focus on adolescents with severe TBI in their study of psychological outcome and agreement between self-ratings and proxy-ratings by “significant others.” Whilst a good degree of agreement was found between patients' and significant others' ratings regarding somatic problems, a lesser degree of agreement was found for nonovert problems such as withdrawal and attention, thought problems, and personal strength.

Moving into adult populations of moderate and severe TBI, T. G. Finnanger et al. evaluated a number of issues regarding executive, emotional, and behavioral problems 2–5 years after injury. This Norwegian neuropsychological study demonstrates significantly more attentional, emotional regulation and psychological difficulties in the TBI group compared to healthy controls. Age, education, traumatic axonal injury, and early depression were important predictors of later executive dysfunctions. This study gives new information to guide the clinical management of TBI survivors.

Most studies of severe TBI have focused on deficits in memory, processing speed, visual spatial abilities, and abstract reasoning. The impact on affective functions as well as awareness during the early stages after brain injury has not been studied to the same extent. A Swedish Icelandic study by M. Stenberg et al. assesses the clinical course of cognitive and emotional impairments in patients with severe TBI from 3 weeks to 1 year after injury and its associations with global and cognitive functioning outcomes at 1 year. Cognition seemed to improve over time and appeared to be rather stable from 3 months to 1 year. Results indicate that early screening of cognitive function could be of importance for rehabilitation planning in a clinical setting.

Health-related quality of life (HRQoL) has only recently gained momentum in the TBI field with novel assessments scales becoming available to be applied to this population. Related to this N. von Steinbuechel et al. from 10 different countries evaluated psychometric performance of a disease-specific QOLIBRI (Quality of Life after Brain Injury) instrument and a generic HRQoL instrument (SF-36) in a large sample of TBI survivors (*N* = 795). QOLIBRI was recommended as the preferable instrument to differentiate between individuals within a health state. QOLIBRI can be an important tool to detect individual recovery patterns after TBI (e.g., disability, depression) and prioritize therapeutic goals.

Few studies have examined sexual functioning in women with TBI, and studies are particularly lacking from regions such as Latin America where a lack of resources for patients with brain injury is well documented. In order to close some of the knowledge gap on this topic, J. Strizzi et al. administered standardized questionnaires which assessed various aspects of sexual functioning, desire, and satisfaction to a group of female TBI survivors and healthy controls from Colombia. Results indicated that nearly all aspects of sexuality are affected after moderate to severe TBI. Researchers also identified a number of unique predictors of poor sexual functioning, suggesting future research directions and rehabilitation interventions strategies.

Mortality and long-term outcomes are assumed to be worse in elderly compared with younger TBI patients. The assumed poor prognosis may influence the treatment strategies applied in older patients. C. Røe et al. evaluate the mortality and functional outcome in old (65–74 years) and very old (>75 years) patients with severe TBI and compare the observed mortality and outcome to the predicted outcome according to the CRASH (Corticosteroid Randomization After Significant Head Injury) models. Results indicated that the CRASH model overestimated mortality and unfavorable outcome in old and very old Norwegian patients with severe TBI. Using such a model in clinical practice may possibly bias treatment decisions in old patients.

In recognition of cultural aspects of TBI, J. Yu et al. reviewed the research and practice in rehabilitation services in Hong Kong. In their exploration of the TBI field they have focused on the Chinese cultural conception of work, stigma associated with TBI, and burden among families. This paper presents a review of 7 studies of rehabilitation trials for brain injured patients in Hong Kong. Existing research shows that rehabilitation services have generally satisfied most of the TBI patients' needs. J. Yu et al. find that future efforts must be diverted to improve vocational rehabilitation and to educate and inform caregivers on the patient's impairments.

In the aftermath of TBI, returning to work (RTW) may be highly desired but is a challenging process for many patients. E. Vikane et al. aimed to identify predictors of RTW 12 months after mild TBI for patients with persistent postconcussion symptoms (PCS) at 6 to 8 weeks of follow-up. Early functional outcomes (i.e., being on sick leave and having disability) together with psychological distress and sick leave during the last year before injury were predictors of RTW. Multidisciplinary outpatient treatment was negatively associated with RTW. Giving much attention to PCS symptoms rather than focusing on aspects concerning RTW may be one exploratory factor for the negative outcome. These findings should be taken into consideration when evaluating future vocational rehabilitation models.

Specific evaluation procedures and methods used by vocational rehabilitation providers have not been studied in depth, and in this special issue C. Dillahunt-Aspillaga et al. from the USA present the results of their online survey of professionals on this topic. The authors discuss their findings in the context of the complexities which individuals with TBI face after injury and argue for evidence-based frameworks of vocational evaluation to be adopted by rehabilitation educators, counselors, vocational evaluators, and other rehabilitation providers.

Many patients who have suffered a TBI require some form of help from caregivers, and it is often relatives who become caregivers. Rehabilitation programs that take caregivers' needs into account have the potential both to promote participation levels for patients and to minimize possible health consequences for the caregivers themselves. L. F. Stevens et al. consider this second aspect, reporting an exploratory study from the USA, of training for caregivers of military service members who have suffered TBI and polytrauma. They find an association between caregiver training and mental health outcomes for caregivers. As the authors suggest, this is an area ripe for future research.
